# Carcinoma of the Colon

**Published:** 1904-04-30

**Authors:** 


### Hospital Clinics and Medical Progress.

### CARCINOMA OF THE COLON.

Mr. H. S. Clogg makes some useful observations on this affection, based upon an analysis of 25 consecutive cases. Amongst these the disease occurred eight times in the cæcal region, three times in the hepatic region, six times in the splenic region, and eight times in the sigmoid flexure and upper rectum. Two distinct types of growth may be recognised. The first involves some three or four inches of bowel, is soft, friable, extensively ulcerated, and does not materially narrow the lumen of the gut. The second type involves a smaller tract of intestine, the lumen of which is almost always diminished. The former is seen most frequently in the cæcal region and not uncommonly in the hepatic; the latter occurs in the recto-sigmoid and splenic regions, and less commonly in the neighbourhood of the hepatic flexure.

Although carcinoma of the colon is a chronic disease, acute or subacute symptoms are frequently present by the time the patient comes under the supervision of the surgeon; this was so in 16 out of the 25 cases under consideration, complete obstruction being present in ten cases, while in six suppuration had occurred. On careful inquiry, however, a suggestive history of indisposition extending over some months may usually be elicited, such as pain, sluggish action of the bowels, deterioration in health, and loss of flesh and energy. Symptoms such as these should never be thought of lightly, since for surgery to attain any considerable measure of success early diagnosis of malignant disease is indispensable. Concerning the occurrence of alternating constipation and diarrhœa, and the passage of blood per rectum, Mr. Clogg remarks that diarrhœa was not present in a single one of the 25 cases, while rectal hæmorrhage was only noticed twice.

In dealing with the question of dissemination, Mr. Cogg controverts the view that in carcimona of the colon the lymphatic glands are only involved late in the course of the disease, and that dissemination by the blood stream, as a rule, occurs earlier than by the lymphatic circulation. He finds that it is usual for the glands to be infiltrated by the time the symptoms are severe enough to bring patients for treatment, while dissemination by the blood channels at this period is uncommon. This is of vital importance when a radical extirpation of the disease is contemplated. It is obvious under the circumstances that excision of the diseased bowel must be accompanied by removal of the nearest chain of lymphatic glands and the lymphatic tract connecting them with the growth, if a hopeful prognosis is to be rendered possible. With a view to discovering how this might be done Mr. Clogg has endeavoured to ascertain which glands should be removed in operating upon cancer situated in the different portions of the colon. He finds that in cancer of the cæcal region the affected glands lie in the angle between the ileum and colon and also for some distance along the ascending colon and in the terminal part of the mesentery. In the region of the hepatic flexure the involved glands form a chain running for some distance along the lower border of the transverse colon. In growths of the splenic flexure the infected glands are situated along the lower border of the transverse colon nearly to its centre. The glands affected from disease of the sigmoid colon and upper rectum are spread out in the mesentery; from them the lymph flow converges to a series of glands situated near the root of the mesentery of the colon. In several cases he has examined the glands which lie along the aorta and vena cava but has never found them enlarged. The conclusion which Mr. Clogg arrives at is that in order to eradicate the affected group of glands more bowel must be taken away than it is the usual custom to remove. Thus in cæcal growths some inches of ileum with its mesentery and nearly the whole of the ascending colon should be removed. In tumour of the hepatic or splenic flexure the corresponding half of the transverse colon with its mesentery should be excised. In recto-sigmoid growths practically the whole loop of bowel must be sacrificed together with its mesentery.

